# Comprehensive Screening for Early Cancer Detection in Individuals With Genetic Predisposition

**DOI:** 10.1200/PO-25-00333

**Published:** 2025-08-20

**Authors:** Natalia A. Bodunova, Airat I. Bilyalov, Anastasiia M. Danishevich, Gurami E. Kvetenadze, Artem K. Andronov, Andrey V. Starshinin, Maria M. Litvinova, Ludmila G. Zhukova, Igor E. Khatkov

**Affiliations:** ^1^ Loginov Moscow Clinical Scientific Center, Moscow, Russia; ^2^ Life Improvement by Future Technologies (LIFT) Center, Moscow, Russia; ^3^ Moscow Health Department, Moscow, Russia; ^4^ Federal State Autonomous Educational Institution of Higher Education I.M. Sechenov First Moscow State Medical University of the Ministry of Health of Russian Federation (Sechenov University), Moscow, Russia

## Abstract

**PURPOSE:**

To evaluate the effectiveness of a comprehensive screening program for individuals with genetic predisposition to cancer, focusing on early detection and improved treatment outcomes through systematic clinical, instrumental, and laboratory monitoring of pathogenic/likely pathogenic (P/LP) variant carriers.

**MATERIALS AND METHODS:**

The registry screening program included two groups: (1) patients with cancer with confirmed germline P/LP variants associated with cancer predisposition, and (2) healthy individuals carrying germline P/LP variants predisposing to cancer development. In total, 816 participants were included. Participants underwent routine screening, including medical history recording, and instrumental and laboratory investigations accompanied by consultations of different specialists. The study compared the age and stage of the disease at the time of cancer diagnosis between healthy P/LP variant carriers (Group A, n = 554) and patients with cancer (Group B, n = 262).

**RESULTS:**

Among the 554 healthy P/LP variant carriers in Group A, 57 patients of cancer (10.2%) were identified, predominantly breast and ovarian cancers (Group A1). In Group A1, 96.4% of detected cancers were at stages I to II. The average age at diagnosis in Group A1 was 52 ± 12 years, with 35% of patients detected within 6 months, 58% within 1 year, and 7% within 1.5 years after inclusion of an individual into the screening program. In Group B1 (191 patients with breast cancer), 20.9% were diagnosed at stages III to IV. Notably, 27% of aggressive molecular biological subtypes of breast cancer (Luminal B and triple-negative) were identified at stage I in Group A1, and the most common *BRCA1* variant was c.5266dupC (rs80357906), accounting for 64.3% of such patients.

**CONCLUSION:**

The comprehensive screening program demonstrated the effectiveness of early detection of malignancies in individuals with genetic predisposition to cancer development. The identification of a significant proportion of early-stage cancers, particularly aggressive subtypes, highlights the importance of tailored screening strategies for the cohort of patients at high risk of cancer development.

## INTRODUCTION

Cancer remains one of the leading causes of morbidity and mortality worldwide, with inherited genetic mutations contributing significantly to the development of certain malignancies.^1-3^ Hereditary cancer syndromes—caused by germline pathogenic or likely pathogenic (P/LP) variants (P/LP)—account for approximately 10% of all cancer patients, and in some cancers, such as breast, ovarian, and colorectal, these mutations may be found in up to 20% of patients.^4,5^ Hereditary cancer syndromes encompass a group of disorders characterized by an increased lifetime risk of malignancies because of germline mutations in cancer susceptibility genes. These syndromes include, among others, hereditary breast and ovarian cancer syndrome, Li-Fraumeni syndrome, Lynch syndrome, and Peutz-Jeghers syndrome.^6^ Recent advances in genetics and molecular diagnostics have improved our understanding of cancer initiation and progression, enabling the development of more personalized approaches to early detection and prevention.^7-9^ These include risk-adapted screening protocols and targeted preventive strategies, which can improve outcomes in high-risk individuals and may ultimately inform population-level cancer control efforts.

**CONTEXT**

**Key Objective**
Can a structured, registry-based screening program improve early cancer detection outcomes in individuals carrying germline pathogenic or likely pathogenic (P/LP) variants in cancer-associated genes?
**Knowledge Generated**
A prospective screening program involving 554 healthy carriers of germline P/LP variants led to the detection of cancer in 10.2% of participants, with 96.4% of patients diagnosed at early stages (I to II). Compared with patients with cancer identified outside the program, screened individuals were significantly more likely to be diagnosed with aggressive subtypes at stage I. These findings support the effectiveness of targeted, genetics-driven screening for improving early detection and clinical outcomes.
**Relevance**
This study provides real-world evidence supporting the implementation of structured screening programs for individuals with hereditary cancer syndromes. The findings underscore the value of precision prevention approaches and can inform public health strategies, policymaking, and clinical practice guidelines aimed at improving cancer outcomes in genetically predisposed populations.

This study specifically addresses individuals with a confirmed genetic predisposition to cancer—namely, carriers of germline P/LP variants in cancer-associated genes such as *BRCA1*, *BRCA2*, *CHEK2*, *PALB2*, *ATM*, *STK11*, and *TP53.* It does not include individuals with a strong family history but without an identified germline mutation.

Effective management of cancer risk in this genetically defined population requires both primary and secondary prevention strategies.^10,11^ Primary prevention aims to stop cancer from developing. It includes educating and counseling on healthy individuals, controlling environmental factors, considering preventive surgeries, and using specific medications to prevent carcinogenesis in the organism. Secondary prevention, through the screening programs, is about detecting of early signs of potential trouble, when treatment is the most effective. These strategies are well established in cohorts of patients with *BRCA*-related breast and ovarian cancers.^12^

Despite strong evidence supporting early detection and risk-reduction strategies, most Russian regions have not put in place systematic follow-up plans for high-risk individuals. The implications of hereditary cancer risk extend far beyond individual patients. According to the rules of the inheritance, in most of patients, children and close relatives of an individual affected by hereditary cancer syndrome have 50% probability of inheriting the genetic cancer predisposition. Although early detection and preventive interventions can dramatically improve outcomes, the current health care system struggles to provide adequate monitoring for these at-risk family members. Multiple systemic barriers contribute to this challenge: health care professionals often demonstrate limited understanding of hereditary cancer predisposition syndromes, there is an insufficient number of genetic counseling specialists, referral guidelines vary significantly between regions, and screening protocols lack standardization across different geographical areas.^13,14^ In addition, although risk-reducing surgical interventions such as mastectomy and salpingo-oophorectomy are recommended by the official clinical guidelines of the Russian Ministry of Health for individuals with clinically significant germline pathogenic variants, their implementation in routine clinical practice remains limited. These procedures are not uniformly reimbursed through the national health care system, and access for healthy carriers may vary depending on regional resources and institutional protocols.

In this report, we present the first results of the Moscow cancer screening program implementation, which included 816 patients with P/LP variants in cancer-associated genes (*BRCA1*, *BRCA2*, *CHEK2*, *PALB2*, *ATM*, *STK11*, and *TP53*). These results are an important step toward organizing of a more comprehensive approach to the prevention of cancer and the early detection of cancer in the cohorts of patients who are genetically predisposed to cancer development.

## MATERIALS AND METHODS

### Study Design and Recruitment

This registry-based screening program was conducted at the Loginov Moscow Clinical Scientific Center and included two distinct groups of participants: Group A—cancer-free carriers: individuals without a personal history of cancer, who were identified as carriers of germline P/LP variants in cancer-associated genes. Group B—patients with cancer: individuals with a confirmed cancer diagnosis (ovarian/breast/pancreatic/endometrial cancer) and a verified germline P/LP variant.

All participants were recruited through the same clinical center and underwent genetic counseling and molecular genetic testing before inclusion in the registry. Testing was initiated on the basis of standardized clinical criteria, including personal and/or family history of cancer, age of onset, and tumor subtype (ovarian/breast/pancreatic/endometrial cancer). All patients were first seen by a clinical geneticist, who determined the indication for testing.

Genetic testing was conducted at the Loginov Moscow Clinical Scientific Center laboratory. Individuals found to carry germline P/LP variants in genes such as *BRCA1*, *BRCA2*, *CHEK2*, *PALB2*, *ATM*, *STK11*, and *TP53* were offered enrollment into the registry program. The patients with cancer were included after confirmation of both malignancy and the genetic mutation. Healthy carriers were enrolled into a prospective follow-up and screening program.

Written informed consent was obtained from all participants before inclusion. The consent form included permission to use depersonalized clinical and genetic data for scientific research and publication.

Statistical analysis was performed using descriptive methods and Fisher's exact test for group comparisons. A *P* value <.05 was considered statistically significant. All analyses were performed using standard statistical software.

### Follow-Up Monitoring

**FIG 1.**
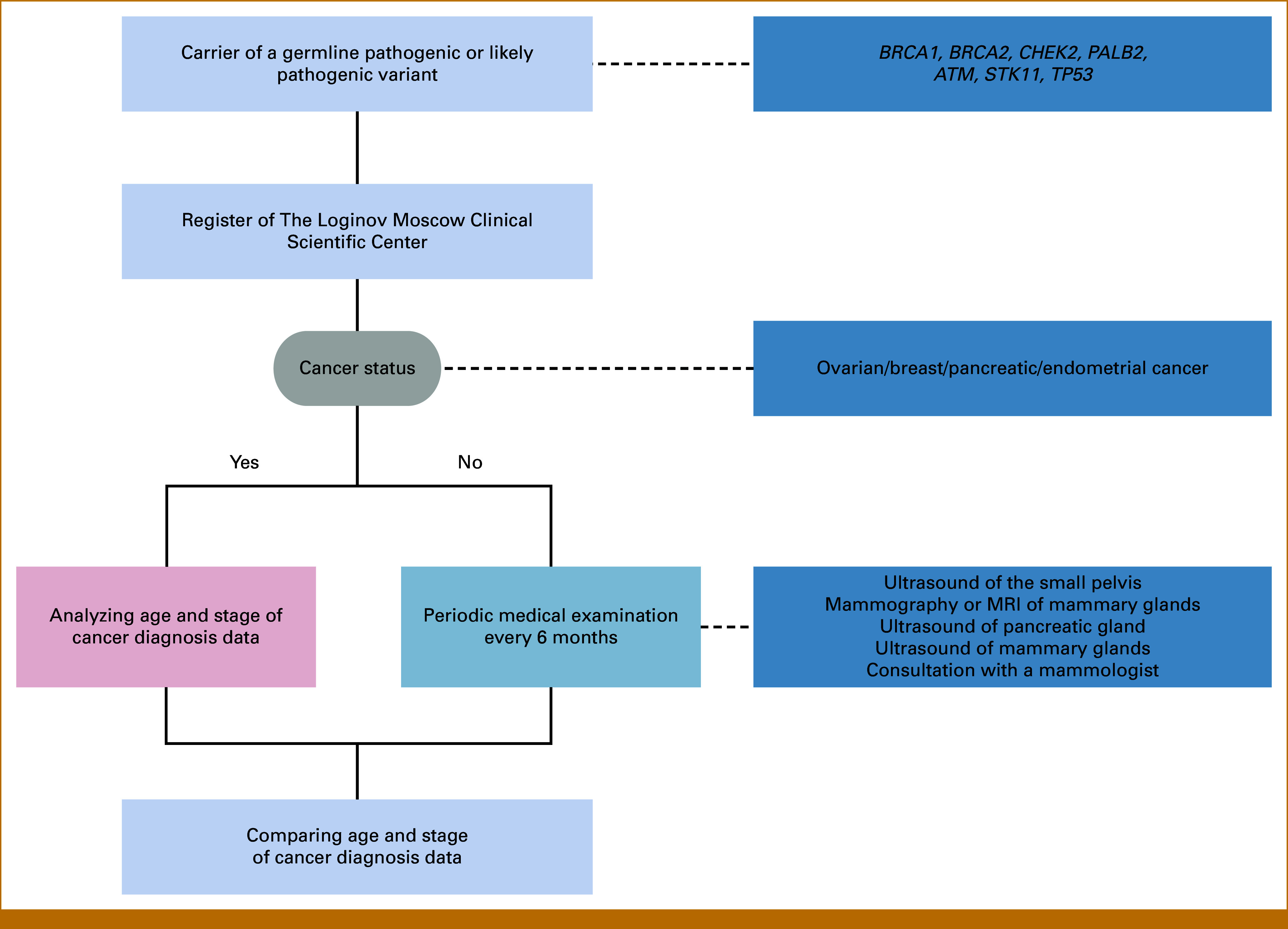
Overview of the study design. MRI, magnetic resonance imaging.

The initial patient examination consisted of a medical history recording, mammologist, gynecologist, and gastroenterologist consultation, and ultrasound examination of the small pelvis organs, mammary glands, and the pancreas together with mammography or magnetic resonance imaging (MRI) of breasts. The results of the analysis were stored in the patient's medical record in the medical database system of the clinic, from where they were transferred to the study database after removal of any personal information. Participants were also invited by phone calls and text messages for a follow-up visit every 6-12 months, depending on the results of previous examinations. At subsequent visits, the examination was repeated with the exception of medical-genetic counseling. The full design is presented in Figure 1.

Participants were enrolled between 2020 and 2024, with ongoing prospective follow-up. Although there was no fixed duration defined at enrollment, patients are intended to be followed longitudinally as part of a continuous screening registry.

### Institutional Review Board Statement

Ethics committee name: The Local Ethics Committee of the Loginov Moscow Clinical Scientific Center. Protocol No.: 921.3 Approval date: 15 February 2022.

## RESULTS

The database of the oncogenetic registry was initiated in 2020. By the end of May 2024, the registry included 816 individuals. Among them, 554 participants (Group A; 49 ± 18 years, 97.5% were females) served as healthy carriers of germline P/LP variant, while 262 (53 ± 14 years, 95% were females) patients had been diagnosed with cancer (Group B). All patients had germline P/LP variant in the genes attributable for hereditary cancer syndromes (genes *BRCA1*, *BRCA2*, *CHEK2*, *PALB2*, *ATM*, *STK11*, and *TP53*). To provide a comprehensive overview of the study population, we present a demographics and mutation profile table for both groups. Table 1 summarizes the distribution of age, sex, and germline P/LP variants in Groups A and B.

**TABLE 1.** Distribution of Germline Pathogenic/Likely Pathogenic Variants in the Study Cohort

Variable	Group A (n = 554)	Group B (n = 262)
Mean age ± SD, years	49 ± 18	53 ± 14
Median age, years	51	44.5
Female, %	97.5	95
*BRCA1*, No. (%)	459 (82.9)	195 (74.4)
*BRCA2*, No. (%)	31 (5.6)	51 (19.5)
*CHEK2*, No. (%)	48 (8.6)	7 (2.7)
*PALB2*, No. (%)	12 (2.2)	6 (2.3)
*ATM*, No. (%)	—	2 (0.8)
*STK11*, No. (%)	4 (0.7)	—
*TP53*, No. (%)	—	1 (0.4)

Abbreviation: SD, standard deviation.

Group A included all healthy individuals carrying germline P/LP variants predisposing to cancer development (n = 554). Among them, a subset (Group A1, n = 57) developed cancer during the follow-up period and was analyzed separately (10.2%), including 56 patients of breast cancer and one patient of ovarian cancer.

The average age at diagnosis in Group A1 was 52 ± 12 years, with a median of 51 years. Among the 57 patients from Group A who were diagnosed with cancer during the screening program, 20 patients (35%) were diagnosed during the first screening visit (within 6 months of enrollment), 33 patients in total (58%) were diagnosed by the second screening visit (within 12 months), and an additional four patients (7%) were diagnosed by the third screening visit (within 18 months of enrollment). The most common P/LP variant of the *BRCA1* gene was c.5266dupC (chr17:g.43057063insG, p.Gln1777ProfsTer74, rs80357906), which accounted for 64.3% of patients (average age 54 ± 12.2 years). Details of all 57 cancer patients, including stage and mutation variant, are presented in the Data Supplement (Table S1).

The following molecular subtypes of breast cancer (Group A1) were identified on the basis of molecular biology of cancer tissue: Luminal A (n = 7, 12.5%), Luminal B (human epidermal growth factor receptor 2 [HER2]–negative; n = 25, 44.6%), triple-negative (n = 23, 41%), and Luminal B (HER2-positive; n = 1, 1.9%). The median Ki-67 value in luminal B was 60%.

The oncogenetic database also included patients who were already diagnosed with cancer at different stages (Group B; 262 patients), including 191 patients of breast cancer (72.9%; Group B1), 58 patients of ovarian cancer (22.14%), 10 patients of pancreatic cancer (3.81%), and 3 patients of endometrial cancer (1.15%).

We focused our comparative analysis on breast cancer patients because they constituted the majority of diagnoses in both groups and allowed for a consistent evaluation of molecular subtypes and staging patterns.

**FIG 2.**
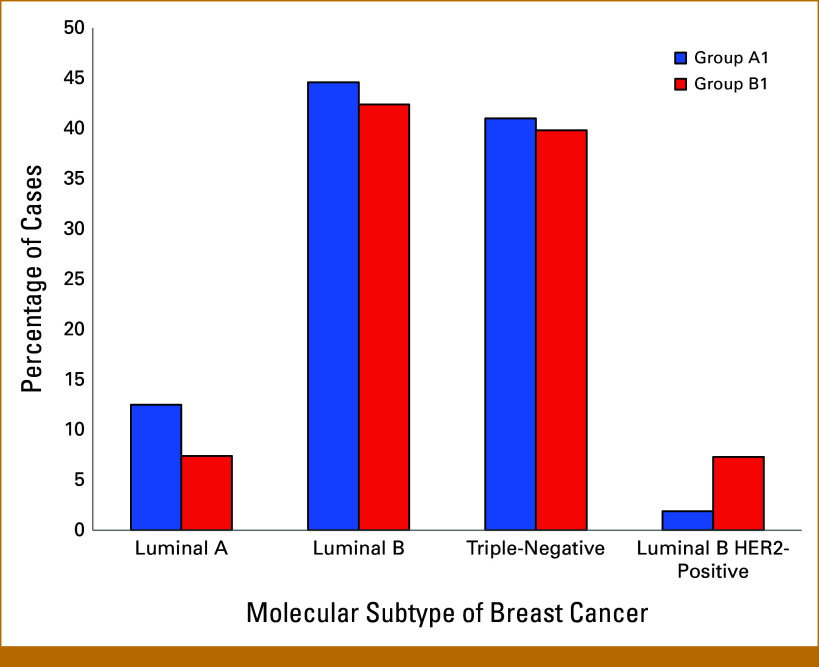
Comparison of breast cancer molecular subtypes between Group A1 and Group B1.

In Group B1, the average age of patients was 44.5 ± 11 years. The molecular subtypes of breast cancer identified were as follows: Luminal A (n = 14, 7.4%), Luminal B (HER2-negative; n = 81, 42.4%), triple-negative (n = 76, 39.8%), Luminal B (HER2-positive; n = 14, 7.3%), and HER2-positive (n = 6, 3.14%). The median Ki-67 value for Luminal B (HER2-negative) was 58.2% (Fig 2). The most prevalent P/LP variant in the *BRCA1* gene was also c.5266dupC (p.Gln1777ProfsTer74), representing 59.1% of the patients (Fig 2).

**FIG 3.**
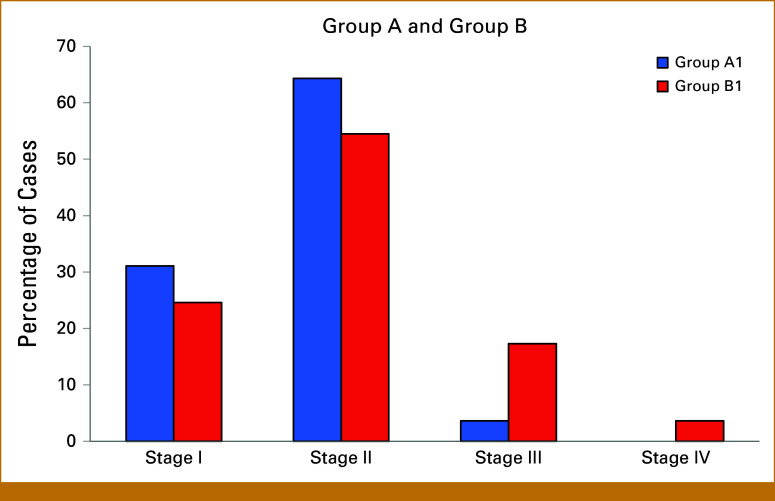
Comparison of cancers stages between Group A1 and Group B1.

Early screening identified in Group A 54 (96.4%) patients of stage I to II and only 2 (3.6%) patients of stage III. Group B exhibited 191 patients of breast cancers, which included 151 (79.1%) stage I to II and 40 (20.9%) patients of stage III to IV. Full details and comparison of groups are presented in Figure 3. Among patients with commonly aggressive breast cancer subtypes (Luminal B, HER2-positive, and triple-negative), 15 of 49 (30.6%) in Group A1 were diagnosed at stage I, compared with only 11 of 113 (9.7%) in Group B1. This difference was statistically significant (*P* = .0019, Fisher's exact test), with an odds ratio of 4.09, indicating a substantially higher likelihood of early-stage detection in the screened population.

The details of the cancer types with the stages and the genes are presented in the Data Supplement (Table S1).

## DISCUSSION

The results of our comprehensive screening program underscore the critical importance of early detection in individuals with genetic predispositions to cancer. In Group A, the identification of 54 (96.4%) patients of stage I to II cancers and only two (3.6%) patients of stage III cancers highlight the efficacy of early screening in detecting malignancies at a stage with a higher chance of cure. Similarly, Group B had 191 patients of breast cancer, including 151 (79.1%) at stages I to II and more than 20% (40) at a more advanced stages III to IV.

The implementation of this screening program allowed to detect 10% of breast cancers within a cohort of P/LP variant carrier patients (group A) during 1.5 years of observation. Our findings indicate a critical need for continuous monitoring of these patients, as the most aggressive breast cancer types, specifically Luminal B and triple-negative, were identified in 27% of patients at stage I by usage of such an approach. Importantly, no metastatic disease was identified in this group. This underscores the importance of tailored screening strategies to enhance early detection and improve treatment outcomes for individuals with genetic predispositions.

Recent studies, including the research by Lowry et al on multimodality screening strategies for women with *BRCA1*/2 variants, reinforce the critical need for targeted screening programs in high-risk populations. These programs have consistently shown that early detection through tailored approaches, such as the combination of digital mammography and MRI, can lead to significant improvements in life expectancy and reductions in breast cancer mortality.^15^

Screening programs for *BRCA1*/2 P/LP variant carriers typically involve a combination of annual MRI and mammography to enhance early detection of breast cancer. Annual MRI screening is particularly recommended because of its high sensitivity, which significantly reduces the incidence of advanced-stage breast cancer in these high-risk individuals.^16,17^ Moreover, recent evidence indicates that adherence to such intensive screening protocols is high after genetic testing and counseling. A study by Naghi et al^18^ demonstrated substantial uptake of breast MRI screening among patients undergoing multiplex gene panel testing, reinforcing the real-world feasibility and acceptance of personalized surveillance strategies in high-risk groups.

The most effective screening strategy identified is alternating MRI with mammography, starting at age 25 years for *BRCA1*/2 P/LP variant carriers, which maximizes life expectancy and minimizes breast cancer mortality.^15^ Additionally, intensive surveillance has been shown to facilitate early diagnosis, leading to improved survival rates after a breast cancer diagnosis.^17^

Establishing a registry of individuals carrying germline P/LP variant is a pivotal step in advancing cancer prevention and early detection efforts. This registry serves as a centralized repository of critical genetic information, facilitating targeted interventions for those at heightened risk. By cataloging this data, health care providers gain a comprehensive understanding of the genetic landscape, enabling them to implement tailored screening and preventative measures.

The introduction of screening programs specifically designed for P/LP variant carriers represents a paradigm shift in cancer care. It acknowledges the unique needs of this high-risk population and provides them with specialized attention. Early identification of potential malignancies through these programs empowers health care providers to intervene at the most opportune moment, vastly improving treatment outcomes.

Importantly, these initiatives have far-reaching implications for patient treatment. Tailored interventions on the basis of genetic predispositions can lead to more effective and personalized therapeutic strategies. This targeted approach may result in earlier diagnoses, enabling less aggressive treatments with potentially fewer side effects. Furthermore, it allows for closer monitoring, facilitating prompt responses to any concerning developments. As a result, patients experience improved quality of care and, ultimately, enhanced overall well-being.

Beyond the direct benefits to patients, the implementation of these programs can yield substantial cost-savings for hospitals and health care systems. The economic benefits of these screening programs include reduced treatment costs because of early diagnosis, which can lead to significant national cost-savings. One study estimated that early diagnosis could save the US health care system approximately $26 billion US dollar (USD).^19^ Additionally, effective screening can lead to mortality reductions in several cancers, thereby decreasing the overall economic burden associated with cancer treatment, which exceeds $157 billion annually in the United States.^20^ Moreover, streamlined and efficient screening programs can optimize resource allocation and reduce unnecessary diagnostic procedures, ultimately contributing to a more cost-effective health care system.^21^

Furthermore, the establishment of detailed registries, as advocated by the WHO, is a pivotal step toward improving the management of genetically determined diseases and screening of carriers. These registries serve as invaluable resources for tracking and monitoring high-risk individuals, enabling more proactive and personalized approaches to cancer prevention and early detection.

At present, there are no publicly available or systematically collected data on the direct cost of breast cancer treatment in the Russian Federation. However, international studies provide clear evidence that early-stage detection significantly reduces treatment expenses. In the United States, first-year treatment costs are approximately $82,121 for stages I to II, while costs for stages III and IV rise to $129,387-$134,682, representing a 57%-64% increase in expenditure for advanced disease.^14^ Similarly, in Germany, treatment of early-stage breast cancer averages €20,284, while stage III to IV treatment costs reach €30,156-€42,086.^22^ In England, the pattern holds: stage I care costs around £5,167, compared with £7,613-£13,330 for more advanced patients.^23^

These consistent findings from multiple health care systems reinforce the economic rationale for shifting cancer diagnoses to earlier stages through structured screening programs, even in countries like Russia where cost data are not yet transparent or centralized.

This study has several limitations. First, all participants were recruited through a single clinical center in Moscow, which may introduce a selection bias toward individuals with access to specialized genetic counseling and care. As such, individuals from rural or socioeconomically disadvantaged regions may be underrepresented. Second, the cohort was predominantly female and of Russian ethnicity, reflecting the high prevalence of *BRCA1/2*-related breast and ovarian cancers, but limiting generalizability to other racial and ethnic groups. Additionally, age distribution was skewed toward middle-aged adults, with relatively few participants younger than 30 years or older than 70 years, which may affect the applicability of findings to these age extremes. Future multicenter studies with broader geographic and demographic inclusion are needed to validate these findings and improve external validity.

In conclusion, empowering precision prevention through targeted screening programs for individuals with a genetic predisposition represents a significant shift in the approach to cancer care. This strategy not only benefits individual patients by enhancing the quality and timeliness of treatment, but also supports broader health care goals by promoting efficient and cost-effective practice. Continued investment in expanding and refining these programs will be critical in the ongoing fight against cancer, leading to improved health outcomes for high-risk populations and cohorts of patients.

## Data Availability

A data sharing statement provided by the authors is available with this article at DOI https://doi.org/10.1200/PO-25-00333.
